# Regression Analysis between the Different Breast Dose Quantities Reported in Digital Mammography and Patient Age, Breast Thickness, and Acquisition Parameters

**DOI:** 10.3390/jimaging8080211

**Published:** 2022-07-31

**Authors:** Salam Dhou, Entesar Dalah, Reda AlGhafeer, Aisha Hamidu, Abdulmunhem Obaideen

**Affiliations:** 1 Department of Computer Science and Engineering, American University of Sharjah, Sharjah P.O. Box 26666, United Arab Emirates; 2 Biomedical Engineering Graduate Program, American University of Sharjah, Sharjah P.O. Box 26666, United Arab Emirates; b00057592@alumni.aus.edu (R.A.); g00087960@aus.edu (A.H.); 3 College of Medicine, Mohammed Bin Rashid University, Dubai P.O. Box 505055, United Arab Emirates; edalah@dha.gov.ae; 4 HQ Diagnostic Imaging Department, Dubai Health Authority, Dubai P.O. Box 4545, United Arab Emirates; 5 Medical Diagnostic Imaging Department, University Hospital Sharjah, Sharjah P.O. Box 72772, United Arab Emirates; abdulmunhem.obaideen@uhs.ae

**Keywords:** radiation dose, full-field digital mammography (FFDM), mean glandular dose (MGD), compressed breast thickness

## Abstract

Breast cancer is the leading cause of cancer death among women worldwide. Screening mammography is considered the primary imaging modality for the early detection of breast cancer. The radiation dose from mammography increases the patients’ risk of radiation-induced cancer. The mean glandular dose (MGD), or the average glandular dose (AGD), provides an estimate of the absorbed dose of radiation by the glandular tissues of a breast. In this paper, MGD is estimated for the craniocaudal (CC) and mediolateral–oblique (MLO) views using entrance skin dose (ESD), X-ray spectrum information, patient age, breast glandularity, and breast thickness. Moreover, a regression analysis is performed to evaluate the impact of mammography acquisition parameters, age, and breast thickness on the estimated MGD and other machine-produced dose quantities, namely, ESD and organ dose (OD). Furthermore, a correlation study is conducted to evaluate the correlation between the ESD and OD, and the estimated MGD per image view. This retrospective study was applied to a dataset of 2035 mammograms corresponding to a cohort of 486 subjects with an age range of 28–86 years who underwent screening mammography examinations. Linear regression metrics were calculated to evaluate the strength of the correlations. The mean (and range) MGD for the CC view was 0.832 (0.110–3.491) mGy and for the MLO view was 0.995 (0.256–2.949) mGy. All the mammography dose quantities strongly correlated with tube exposure (mAs): ESD (R^2^ = 0.938 for the CC view and R^2^ = 0.945 for the MLO view), OD (R^2^ = 0.969 for the CC view and R^2^ = 0.983 for the MLO view), and MGD (R^2^ = 0.980 for the CC view and R^2^ = 0.972 for the MLO view). Breast thickness showed a better correlation with all the mammography dose quantities than patient age, which showed a poor correlation. Moreover, a strong correlation was found between the calculated MGD and both the ESD (R^2^ = 0.929 for the CC view and R^2^ = 0.914 for the MLO view) and OD (R^2^ = 0.971 for the CC view and R^2^ = 0.972 for the MLO view). Furthermore, it was found that the MLO scan views yield a slightly higher dose compared to CC scan views. It was also found that the glandular absorbed dose is more dependent on glandularity than size. Despite being more reflective of the dose absorbed by the glandular tissue than OD and ESD, MGD is considered labor-intensive and time-consuming to estimate.

## 1. Introduction

Breast cancer is reported as one of the leading causes of mortality for women worldwide [1]. It has surpassed lung cancer as the most commonly diagnosed cancer worldwide, according to the GLOBOCAN 2020 estimates of cancer incidence and mortality produced by the International Agency for Research on Cancer [2]. It is the leading cause of cancer death among women worldwide. Breast cancer is the most common cancer among female United Arab Emirates (UAE) citizens (32.16%) and non-UAE citizens (41.41%) [3]. To date, there is no definite known cause of breast cancer or an effective method to prevent it.

Thus far, mammography is considered the most effective means of early detection of breast cancer [4]. With this, the potential risk of radiation-induced carcinogenesis in some high-risk patients is also increasing, leading to the possibility of limiting the number of screening mammograms or deterring women from undergoing breast screening [5]. Mammography images are obtained using a low-energy X-ray radiation beam [6]. Exposure to such low radiation repeatedly can lead to an adverse impact known as the radiation stochastic effect [1]. The glandular tissue of the breast is considered one of the most radiosensitive organs in the body [7], which makes optimizing equipment performance and managing the radiation dose per each mammogram exam a necessity that cannot be overemphasized to ensure the ”as low as reasonably achievable (ALARA)” radiation dose principle.

Mammography mean glandular dose (MGD), which is used synonymously with average glandular dose (AGD), has been widely accepted as the most appropriate measurement for predicting the risk of radiation-induced cancer [8]. Most advanced mammography units provide a means to estimate the MGD or AGD for each patient, where several different algorithms exist and are used by each manufacturer. Thus, in addition to mammography exposure, known as the entrance skin dose (ESD), and the half-value layer (HVL) dedicated for X-ray (target and filter type) spectra, breast thickness and breast density are considered key inputs for such algorithms.

In addition to the ESD dose quantity reported by the mammography machine, organ dose (OD) is another dose quantity that is reported by mammography machines. However, MGD and OD are not the same. A strong significant difference between the MGD and OD was reported in [9].

Thus, it is important to have a good understanding and an accurate estimation of the parameters that can affect the glandular absorbed dose, as it provides an indication of the radiation risk to the breast during exposure. The objectives of this paper are as follows:

- Estimate the MGD per view, breast thickness, and age group for every subject enrolled in this study,
- Evaluate the impacts of mammography acquisition parameters, age, and breast thickness on the estimated MGD and other machine-produced dose quantities using a multilinear regression model,
- Conduct a correlation study between the ESD and OD, and the estimated MGD for each image view,
- Compare the findings of this study with other studies conducted on samples selected from different demographic regions.

## 2. Materials and Methods

### 2.1. Mammography Data

The proposed study was applied on breast screening mammogram images captured from the UAE population. All mammography screening examinations were performed using a Siemens Mammomat Inspiration (Siemens Medical Solutions, Forchheim, Germany). A dataset of 2035 mammograms from a cohort of 486 subjects with an age range of (28–86 years) who underwent screening mammography examinations was collected from the University Hospital of Sharjah, UAE, and retrospectively analyzed. This study was approved by the Institutional Review Board (IRB) committee of our university and hospital ethics committees. Mammography acquisition parameters, including image view, number of views, target and filter combination, compressed breast thickness, ESD, organ dose, and other acquisition parameters (kVp and mAs), for all the patients were extracted from the digital imaging and communications in medicine (DICOM) headers and used in this study. The HVL for our Tungsten (W)/Rhodium (Rh) spectra was 0.45 mm Al. A dedicated g conversion factor for each view was obtained from [10,11] for each subject based on the HVL (herein = 0.45 mm Al) and breast thickness per view of each subject.

### 2.2. Estimating Mean Glandular Dose (MGD)

The MGD was estimated per view, breast thickness, and age group for every subject enrolled in this study using Dance et al.’s method [10,11]. The ESD dose, measured in mGy for mammography views such as craniocaudal (CC), medio-lateral oblique (MLO), medio-lateral (ML), and lateromedial (LM), for each and every subject involved in this study was input into the mathematical model presented in Equation (1), as discussed in [10]:

(1) $$MGD\left(mGy\right)=K\;\cdot \;g\;\cdot \;c\;\cdot \;s$$

where

- K is the mammography machine output (calibration) measured in mGy. It is also known as the entrance dose at the surface of the breast. This quantity was provided by the manufacturers for each mammography scan and could also be obtained from the DICOM header.
- g is a conversion factor describing the fraction of “K” that is absorbed by the glandular tissue in the breast. g depends on breast thickness and the HVL.
- c is a correction factor for breast composition that corrects for any difference in glandularity from 50%, i.e., from 0–100%. Dance et al. [10] provided a reference table of c factors for various HVLs, breast thicknesses from (2–11) cm, and glandularity from which one can extrapolate the percentage of glandularity for each individual.
- s is a correction factor for the X-ray spectrum that can be altered when using different target and filter combinations. Such a correction factor is independent of the HVL and can be found in [10] in a simple reference table that includes various target and filter combinations.

### 2.3. Regression Analysis

Multilinear regression models were built to quantify the impacts of mammography acquisition parameters, age, and breast thickness as input parameters on output parameters related to the mammographic organ dose. A linear equation representing the regression model assigns a scale factor (called the coefficient) to each input parameter, with one additional coefficient (called the intercept) added to the equation. The input parameters considered in this work were the age of the patient, the compressed breast thickness, and the acquisition parameters, namely, X-ray tube voltage (kVp), exposure time (msec), exposure (mAs), and X-ray tube current (mA). The output parameters considered in this work were the entrance skin dose (ESD) and organ dose (OD), which were reported after each mammography scan in addition to the estimated MGD, as described in Equation (1). A regression model was estimated for output parameters individually. The multilinear regression model could be represented as a function of the input and output parameters, as follows:

(2) $$\hat{Y}=\beta_{0}+\beta_{1}\cdot I_{1}+\beta_{2}\cdot I_{2}+\ldots +\beta_{n}\cdot I_{n}$$

where $\hat{Y}$ is the estimated output parameter (dose quantity) using the regression model; $I_{1},\;I_{2},\;\ldots ,\;I_{n}$ are the input parameters; and $\beta_{0},\;\beta_{1},\;\ldots ,\;\beta_{n}$ are the coefficients (scaler factors). To estimate the values of the coefficients, a least-squares method was used.

Moreover, the individual correlations between each of the input parameters and each output parameter (dose quantity) were assessed by fitting each pair of these input and output parameters to a linear regression model, as shown in the equation below:

(3) $$\hat{Y}=\beta_{0}+\beta_{1}\cdot I_{j}$$

where $I_{j}$ is one input parameter selected from the set of input parameters $I_{1},\;I_{2},\;\ldots ,\;I_{n}$.

The correlations between different mammography dose quantities, namely, machine-provided OD and ESD and calculated MGD, were also investigated. To achieve this purpose, the individual correlations between these dose quantities were assessed by fitting each pair of them to a regression model similar to what was proposed in Equation (3).

### 2.4. Regression Evaluation

The regression models were evaluated using different criteria, such as the coefficient of determination (*R*^2^), mean square error (*MSE*), mean absolute error (*MAE*), and mean absolute percentage error (*MAPE*). The coefficient of determination, or R-squared ($R^{2}$), is a metric to assess the goodness-of-fit for linear regression models. This metric measures the strength of the relationship between a fitted model and an output parameter on a 0–1 scale, where 0 represents no relationship, and 1 represents a strong relationship. $R^{2}$ can be calculated as follows:

(4) $$R^{2}=1-\frac{\sum_{i=1}^{N}{\left(Y_{i}-{\hat{Y}}_{i}\right)}^{2}}{\sum_{i=1}^{N}{\left(Y_{i}-{\bar{Y}}_{i}\right)}^{2}}$$

where ${\bar{Y}}_{i}$ is the mean of the $Y_{i}$ values.

MSE, MAE, and MAPE are calculated as follows:

(5) $$MSE=\frac{\sum_{i=1}^{N}{\left(Y_{i}-{\hat{Y}}_{i}\right)}^{2}}{N}$$

(6) $$MAE=\frac{1}{N}\;\overset{N}{\underset{i=1}{\sum }}\left|Y_{i}-{\hat{Y}}_{i}\right|$$

(7) $$MAPE=\frac{1}{N}\;\overset{N}{\underset{i=1}{\sum }}\left|\frac{Y_{i}-{\hat{Y}}_{i}}{Y_{i}}\right|\times 100\%$$

where N is the number of data samples, and Y is the output parameter reported after each mammography scan, i.e., ESD or OD, or estimated, i.e., MGD, as in Equation (1). The MSE, MAE, and MAPE metrics were all applied by comparing the predicted output parameter using the regression model with the reported or estimated parameter Y.

## 3. Results

### 3.1. Mammography Data and MGD Estimation

In this section, the mammography data are described, along with the machine-reported and calculated dose quantities. Table 1 shows a descriptive summary of different dose quantities, namely, ESD, OD, and MGD, for each subject enrolled in this study based on image view, with the dominant views being CC and MLO.

Table 2 reports ESD, OD, and calculated MGD based on breast thickness. Here, we adapted the classification reported in [12] of fatty for a breast thickness of 5–7 cm, medium for a breast thickness 3–5 cm, and dense for a 2–3 cm breast thickness. Table 3 reports the entrance dose, organ dose and calculated MGD based on age ranges.

### 3.2. Results of the Regression Analysis

In this section, the regression models that were built using the mammography data to quantify the impacts of mammography acquisition parameters and patient information, such as age and breast thickness, on mammographic dose quantities are evaluated. Table 4 shows the results of evaluating the regression models between the acquisition parameters and the dose quantities, namely, ESD, OD, and MGD, for each view using the regression evaluation criteria introduced in Section 2.4.

Moreover, the correlations between MGD and both ESD and OD are investigated in this work. Table 5 shows the regression metrics evaluation between MGD and both ESD and OD for each view.

## 4. Discussion

MGD has been widely accepted as the most appropriate measure for estimating the radiation-induced risk of breast cancer [8]. It is important to mention that MGD is a calculated dose index, meaning it is not automatically prompted by commercial mammography machines. Hence, it is only logical to assume that MGD accuracy depends on the method used for calculation. Salomon et al. [13] conducted a study to compare between three different methods, including Wu et al. [14] Dance et al. [10,11] and Volpara [15]. While Volpara provided a tissue composition analysis allowing for a more accurate glandularity estimation, the Wu and Dance methods both used a reference table and customized conversion factors. Nevertheless, recent studies still report MGD using the Dance method, including [9,12,16], similar to what was performed in this study.

Mammography exams tend to vary in the number of views performed, as well as the dose per view, resulting in wide variation in the MGDs absorbed amongst individuals. The American College of Radiology Imaging Network (ACRIN) Digital Mammographic Imaging Screening Trial (DMIST) reported an average MGD of 3.7 mGy from two-view digital mammography (1.86 mGy per view) [17]. Herein, Table 1 demonstrated diverse MGDs across the cohort of subjects enrolled, representing a sample from the UAE population. The mean ± SD (and range) MGD for the CC view was 0.832 ± 0.296 (0.11–3.49) mGy and for the MLO view was 0.995 ± 0.350 (0.26–2.95) mGy. While small differences were seen between the mean MGDs of the CC and MLO views, MLO yielded the high-end dose. Such findings agree with other studies in the field [12,16,18,19]. Jamal et al. [18] reported mean MGDs in a sample of Malaysian subjects of 1.54 mGy and 1.82 mGy for the CC and MLO views, respectively. Chevalier et al. [19] reported a mean (and range) MGD of 1.80 (0.4–6.9) mGy for the CC view and 1.95 (0.6–8.1) mGy for the MLO view. Similarly, Al Naemi et al. [12] reported a mean (and range) MGDs based on views measured in Qatari subjects as 1.90 (0.8–6.16) mGy for the CC view and 1.97 (0.7–6.13) mGy for the MLO view. An average MGD of 0.74 mGy and ranges (0.33 to 6.41) mGy and (0.28 to 8.59) mGy for the CC and MLO views, respectively, representing a cohort sample from Nigeria, were reported by Josephine and colleagues [16].

Breast compression seemed to contribute to MGD. Table 4 showed a positive moderate correlation between the average MGD and the compressed breast thickness (R^2^ = 0.315 and 0.351 for the CC and MLO views, respectively). The positive correlation between MGD and compressed breast thickness demonstrated in the present study has been illustrated in previous studies. Riabi et al. [20] showed a significant correlation between MGD and compressed breast thickness with a correlation coefficient of 0.692. Analyzing the difference between the MGDs of thin breasts (<5 cm) and thick breasts (>5 cm), a significant difference was seen (*p* < 0.01). Du et al. [21] also reported a positive correlation between MGD and compressed breast thickness. Chevalier et al. [19] analyzed the doses of 5034 patients who had undergone full-field digital mammography and concluded that differences between the corresponding MGDs among all the groups were lowest for thin breasts, with a mean ± standard deviation (SD) breast size of 34 ± 8 mm, compared to breast sizes of 61 ± 11 mm. This observation is in line with our reported findings presented in Table 2. Fatty breasts measuring (5–7) cm yielded a higher mean (and range) MGD than dense breasts measuring (2–3) cm, indicating that large-breast-size women tend to receive higher doses per view and may require more than four views for complete examination [5]. In contrast, Al Naemi et al. [12] reported a reverse observation with the mean (and range) MGD decreasing in fatty breasts measuring (5–7) cm and increasing in dense breasts measuring (2–3) cm.

Age also seemed to be a factor that could impact MGD, as it was associated with glandularity and density. Herein, we observed a lower MGD for subjects 64 years and older. Similar findings were reported by Pwamang et al. [1], where patients with a compressed breast thickness of 32 mm in the age group of 40–49 years reported an MGD of 1.55 mGy, whereas a compressed breast thickness of 60 mm in the age group of 50–64 had an MGD of 2.51 mGy. Chevalier et al. [19] reported a mean ± SD MGD of 1.85 ± 0.01 for subjects younger than 50 years and 1.90 ± 0.01 for those above 50 years. On the other hand, Baek et al.’s [22] correlation and regression study concluded that age was negatively associated with MGD (*p* < 0.05).

Image acquisition parameters such as kVp and mAs seemed also to impact the resulting MGDs, with X-ray tube current showing a substantial impact comparing to tube voltage (kVp). Strong correlation (R^2^ = 0.980 and R^2^ = 0.972 for CC and MLO, respectively) were seen between the MGDs and the mAs. Weak correlation (R^2^ = 0.292 and 0.304 for CC and MLO, respectively) were also seen between the MGDs and the applied kVp. These results are in agreement with the results reported by Riabi et al. [20], where a statistically significant correlation (*p* < 0.01) was seen between the MGD and each of the applied kVp and mAs amounts, with a correlation coefficient of 0.829 between MGD and kVp and of 0.890 between MGD and mAs.

Baek et al. [22] conducted a multivariate linear regression analysis to investigate the impacts of mammographic composition and breast size on the glandular dose during full-field digital mammography (FFDM) in Korean women. Using a multivariate linear regression analysis, they found that the mAs, kVp, compressed breast thickness, and mammographic breast size were positively associated with MGD (*p* < 0.05). Patients with radiation dose values above the diagnostic reference value had large breasts of dense composition.

Table 5 showed that there was a strong correlation between the calculated MGD and the ESD and OD values reported by the mammogram unit: ESD (R^2^ = 0.929 for the CC view and R^2^ = 0.914 for the MLO view) and OD (R^2^ = 0.971 for the CC view and R^2^ = 0.972 for the MLO view). This also showed that OD had a stronger correlation with MGD despite the strong significant difference between MGD and OD that was reported by Suleiman et al. [9].

To our knowledge, limited studies have investigated the correlation between OD and MGD, with most studies focusing only on ESD and MGD [16,21]. Using reference tables to estimate MGD is time-consuming and incorporates uncertainties [13]. Breast density seems to be a significant contributing parameter, directly influencing breast cancer detection accuracy [23]. Denser breasts indirectly impact the MGD, as the noise level associated with dense breasts is higher [24]. Some limitations were encountered in the present study. Primarily, mammography image quality evaluations were not part of this study. We assumed the mammography images obtained were all diagnostically adequate. Further, the data obtained were restricted to a single healthcare institute and a single mammography machine. A larger sample size is needed in order to establish an average MGD baseline for the UAE population. Moreover, breast density evaluations were not part of the study. In addition, the MGDs for the enrolled subjects were estimated based on Dance et al.’s [10,11] bulk reference tables.

## 5. Conclusions

This study proposes an estimation and good understanding of parameters that could affect the glandular absorbed dose. The MGD was estimated per view, breast thickness, and age group for every subject enrolled in this study. Moreover, an evaluation of the impacts of mammography acquisition parameters, age, and breast thickness on the estimated MGDs and other machine-produced dose quantities using a multilinear regression model was conducted. Furthermore, a correlation study between the ESD and OD, as well as the estimated MGD, for each image view was discussed. This retrospective study was conducted on mammography scan data for subjects who underwent screening mammography examinations. The findings of this study were compared to the findings of other studies conducted on samples selected from different demographic regions. It was found that the mean MGD for the MLO view was slightly higher than that of the CC view. The findings of the regression analysis showed that all the mammography dose quantities, namely ESD, OD, and MGD, were strongly correlated with tube exposure (mAs). However, patient age showed poor correlation with all the mammography dose quantities. Breast thickness showed better correlation with all the mammography dose quantities compared to patient age. Moreover, it was found that there was a strong correlation between the calculated MGD and the ESD and OD values reported by the mammogram unit, with OD showing a stronger correlation with MGD.

## Figures and Tables

**Table 1:** Descriptive summary of entrance skin dose (ESD), organ dose (OD), and mean glandular dose (MGD) based on view.

View	Number of Images	Average	Standard Deviation	Range (Min–Max)
**Entrance Skin Dose (mGy)**				
CC	1013	3.698	1.763	0.452–21.768
MLO	1013	4.996	2.356	1.047–18.730
**Organ Dose (mGy)**				
CC	1013	0.012	0.004	0.002–0.045
MLO	1013	0.014	0.005	0.003–0.038
**Mean Glandular Dose (mGy)**				
CC	1013	0.832	0.296	0.110–3.491
MLO	1013	0.995	0.350	0.256–2.949

**Table 2:** Descriptive summary of entrance skin dose (ESD), organ dose (OD), and mean glandular dose (MGD) based on breast thickness.

Compressed Breast Thickness (cm)	Number of Images	Average	Standard Deviation	Range (Min–Max)
**Entrance Skin Dose (mGy)**				
Fatty (5–7)	1290	4.122	1.405	0.452–11.372
Medium (3–5)	346	2.351	0.740	0.940–5.201
Dense (2–3)	12	1.414	0.333	0.824–1.952
**Organ Dose (mGy)**				
Fatty (5–7)	1290	0.012	0.004	0.002–0.031
Medium (3–5)	346	0.010	0.003	0.005–0.019
Dense (2–3)	12	0.009	0.002	0.005–0.011
**Mean Glandular Dose (mGy)**				
Fatty (5–7)	1290	0.898	0.268	0.110–2.436
Medium (3–5)	346	0.646	0.178	0.317–1.318
Dense (2–3)	12	0.529	0.105	0.323–0.696

**Table 3:** Descriptive summary of entrance skin dose (ESD), organ dose (OD), and mean glandular dose (MGD) based on age group.

Age Group (Years)	Number of Images	Average	Standard Deviation	Range (Min–Max)
**Entrance Skin Dose (mGy)**				
(Less than 40) years	203	4.609	2.745	1.415–21.768
(40–49) years	910	4.656	2.204	0.452–17.769
(50–64) years	793	4.016	1.827	1.095–14.462
(Older than 64) years	129	3.842	2.539	0.824–16.260
**Organ Dose (mGy)**				
(Less than 40) years	203	0.014	0.006	0.007–0.045
(40–49) years	910	0.014	0.004	0.002–0.032
(50–64) years	793	0.012	0.004	0.003–0.032
(Older than 64) years	129	0.011	0.005	0.005–0.034
**Mean Glandular Dose (mGy)**				
(Less than 40) years	203	0.995	0.433	0.483–3.491
(40–49) years	910	0.945	0.322	0.110–2.375
(50–64) years	793	0.865	0.291	0.252–2.460
(Older than 64) years	129	0.845	0.416	0.317–2.699

**Table 4:** Evaluation of regression analyses between patient age, compressed breast thickness, and acquisition parameters as input parameters and entrance skin dose (ESD), organ dose (OD), and mean glandular dose (MGD) as output parameters for each view.

Output Parameter: Entrance Skin Dose (ESD)								
Input Parameters	R^2^		MSE		MAE		MAPE	
	CC	MLO	CC	MLO	CC	MLO	CC	MLO
Patient age	0.053	0.030	2.942	5.376	1.213	1.727	0.385	0.429
Compressed breast thickness (cm)	0.499	0.568	1.555	2.398	0.884	1.139	0.255	0.248
X-ray tube voltage (kVp)	0.454	0.492	1.696	2.819	0.918	1.214	0.262	0.257
Exposure time (msec)	0.816	0.962	0.570	0.214	0.430	0.357	0.161	0.099
Exposure (mAs)	0.938	0.945	0.194	0.306	0.314	0.413	0.087	0.086
X-ray tube current (mA)	0.257	0.085	2.307	5.073	0.976	1.680	0.259	0.371
All input parameters (listed above)	0.989	0.999	0.033	0.006	0.105	0.056	0.032	0.013
Subset of highly correlated parameters (exposure time and exposure)	0.945	0.962	0.170	0.211	0.304	0.359	0.087	0.095
**Output Parameter: Organ Dose (OD)**								
Patient age	0.096	0.066	1.37 × 10^−5^	2.06 × 10^−5^	0.0027	0.0034	0.252	0.265
Compressed breast thickness (cm)	0.217	0.294	1.19 × 10^−5^	1.55 × 10^−5^	0.0026	0.0030	0.243	0.231
X-ray tube voltage (kVp)	0.204	0.257	1.21 × 10^−5^	1.64 × 10^−5^	0.0027	0.0031	0.242	0.231
Exposure time (msec)	0.827	0.951	2.63 × 10^−6^	1.07 × 10^−6^	0.0010	0.0007	0.102	0.063
Exposure (mAs)	0.969	0.983	4.69 × 10^−7^	3.69 × 10^−7^	0.0004	0.0004	0.041	0.035
X-ray tube current (mA)	0.325	0.154	1.02 × 10^−5^	1.86 × 10^−5^	0.0021	0.0033	0.180	0.236
All input parameters	0.995	0.996	6.92 × 10^−8^	7.92 × 10^−8^	0.0001	0.0002	0.014	0.015
Subset of highly correlated parameters (exposure time and exposure)	0.974	0.984	3.97 × 10^−7^	3.53 × 10^−7^	0.0005	0.0005	0.042	0.036
**Output Parameter: Mean Glandular Dose (MGD)**								
Patient age	0.054	0.032	0.083	0.118	0.211	0.260	0.276	0.284
Compressed breast thickness (cm)	0.315	0.351	0.060	0.079	0.183	0.210	0.233	0.221
X-ray tube voltage (kVp)	0.292	0.304	0.062	0.085	0.187	0.217	0.234	0.224
Exposure time (msec)	0.814	0.946	0.016	0.007	0.074	0.060	0.110	0.071
Exposure (mAs)	0.980	0.972	0.002	0.003	0.030	0.043	0.037	0.043
X-ray tube current (mA)	0.333	0.147	0.058	0.104	0.163	0.241	0.187	0.244
All input parameters	0.990	0.980	0.001	0.003	0.020	0.037	0.025	0.037
Subset of highly correlated parameters (exposure time and exposure)	0.982	0.973	0.002	0.003	0.030	0.043	0.038	0.043

Coefficient of determination (R^2^) values closer to 0 indicate no relationship and closer to 1 indicate a strong relationship (correlation). Mean absolute error (MAE) values closer to 0 indicate a higher correlation (i.e., perfect model); mean square error (MSE) is always bigger than MAE due to squaring the difference; mean absolute percentage error (MAPE) is the percentage equivalent to the MAE.

**Table 5:** Evaluation of regression analysis between entrance skin dose (ESD) and organ dose (OD) as input parameters and mean glandular dose (MGD) for each view.

Output Parameter: Mean Glandular Dose (MGD)								
Input Parameters	R^2^		MSE		MAE		MAPE	
	CC	MLO	CC	MLO	CC	MLO	CC	MLO
ESD	0.929	0.914	0.006	0.011	0.057	0.074	0.071	0.075
OD	0.971	0.972	0.003	0.003	0.039	0.046	0.049	0.048

Coefficient of determination (R^2^) values closer 0 indicate no relationship and closer to 1 indicate a strong relationship (correlation). Mean absolute error (MAE) values closer to 0 indicate a higher correlation (i.e., perfect model); mean square error (MSE) is always be bigger than MAE due to squaring the difference; mean absolute percentage error (MAPE) is the percentage equivalent to the MAE.

## Data Availability

Not applicable.
